# Clutter Suppression for Indoor Self-Localization Systems by Iteratively Reweighted Low-Rank Plus Sparse Recovery

**DOI:** 10.3390/s21206842

**Published:** 2021-10-14

**Authors:** Jesús Sánchez-Pastor, Udaya S. K. P. Miriya Thanthrige, Furkan Ilgac, Alejandro Jiménez-Sáez, Peter Jung, Aydin Sezgin, Rolf Jakoby

**Affiliations:** 1 Institute of Microwave Engineering and Photonics, Technical University of Darmstadt, 64283 Darmstadt, Germany; alejandro.jimenez_saez@tu-darmstadt.de (A.J.-S.); rolf.jakoby@tu-darmstadt.de (R.J.); 2 Institute of Digital Communication Systems, Ruhr University Bochum, 44801 Bochum, Germany; furkan.ilgac@rub.de (F.I.); aydin.sezgin@rub.de (A.S.); 3 Institute of Communications and Information Theory, Technical University Berlin, 10587 Berlin, Germany; peter.jung@tu-berlin.de; 4 Data Science in Earth Observation, Technical University of Munich, 82024 Taufkirchen/Ottobrunn, Germany

**Keywords:** chipless radio-frequency localization (RFID), indoor self-localization, clutter separation, low-rank, rpca, weighted norm

## Abstract

Self-localization based on passive RFID-based has many potential applications. One of the main challenges it faces is the suppression of the reflected signals from unwanted objects (i.e., clutter). Typically, the clutter echoes are much stronger than the backscattered signals of the passive tag landmarks used in such scenarios. Therefore, successful tag detection can be very challenging. We consider two types of tags, namely low-Q and high-Q tags. The high-Q tag features a sparse frequency response, whereas the low-Q tag presents a broad frequency response. Further, the clutter usually showcases a short-lived response. In this work, we propose an iterative algorithm based on a low-rank plus sparse recovery approach (RPCA) to mitigate clutter and retrieve the landmark response. In addition to that, we compare the proposed approach with the well-known time-gating technique. It turns out that RPCA outperforms significantly time-gating for low-Q tags, achieving clutter suppression and tag identification when clutter encroaches on the time-gating window span, whereas it also increases the backscattered power at resonance by approximately 12 dB at 80 cm for high-Q tags. Altogether, RPCA seems a promising approach to improve the identification of passive indoor self-localization tag landmarks.

## 1. Introduction

Indoor localization has potential applications in many sectors such as health, surveillance, building management, robotics, and many more [1]. In an indoor self-localization system, where an autonomous mobile robot or transducer can calculate its own position and establish itself in a common coordinate system, reference positions are needed, henceforth indistinctly referred to as landmarks or tags. They can be classified as active or passive, depending on whether they interact proactively in the communication link or scatter back the interrogating wave sent by a mono-static radar.

On the one hand, active landmarks include Wi-Fi hot-spots [2,3] or active RFID tags [4]. However, they fall short in harsh environments where very high temperatures, powerful vibrations or high pressure might make state-of-the-art devices lose their precision and reliability [5]. On the other hand, passive RFID tags are promising to operate in such problematic environments, for instance, by being manufactured using novel materials such as ceramics. Although they showcase lower ranges than their active counterparts, owing to the lack of a powered supply, they can be considered as *cooperative radar targets* that, without actively participating on the communication link, reflect a wave back to a reader. In this direction, several tags have been developed over recent years [3,6,7,8,9]. Assuming that these passive tags are located at fixed positions, and by detecting several of them, a reader can estimate its own position inside a common Cartesian coordinate system by time-of-flight and trilateration techniques [10].

One of the main challenges of passive RFID-based localization is the suppression of the reflected signals from unwanted objects (i.e., clutter). Typically, the reflected signal amplitude of the clutter is much larger than the landmarks’ backscattered responses. Therefore, it might mask the desired signal, resulting in a very challenging landmark detection. This effect is exacerbated for higher frequencies [11], which is extremely important for high-precision self-localization systems, as the localization precision scales up with frequency. Thus, sophisticated signal separation methods are required to separate the backscattered response of the tag from the clutter.

Widely known state-of-the-art clutter suppression methods are subspace projection (SP) [12], spatial filtering (SF), [13] and time-gating. However, they face several challenges. In SP, it is required to determine the perfect threshold for clutter suppression, which typically cannot be established easily. Also, in time-gating, it is required to determine the time interval in which the backscattered response of the tag expands. Its accurate estimation determines the success of clutter removal, which is very difficult to do when a clutter source and tag are located in close proximity. Thus, in this work, we propose a low-rank plus sparse recovery approach for clutter removal. Here, we profit from the low-rankness and sparseness properties of the responses of the tag and clutter. Note that in this work, we consider two types of tags based on their Q-factor, namely low-Q and high-Q tags, depending on whether their temporal response is short-lived or presents long ringing time, respectively.

The landmark tags exhibit several properties in their time domain and frequency domain responses that can be used to apply the aforementioned clutter-separation algorithm. On the one hand, the backscattered response of the high-Q tag is a broad pulse in the time domain, which is sparse in the frequency domain. Hence, the Hankel matrix generated by the time domain backscattered response of the high-Q tag is a low-rank matrix [14]. On the other hand, the signalling response of clutter is sparse compared to the high-Q tag’s response in the time domain. Therefore, the low-rank plus sparse recovery approach is proposed to identify the high-Q tag’s backscattered response from clutter. Note that due to the complementary responses of low-Q tag and high-Q tag, the same low-rank and sparse concept is used to identify the response of the former with the following modification.

In the low-Q tag scenario, the backscattered response is short in time domain and hence sparse. As aforementioned, the signaling response of the clutter is sparse in the time domain as well. In this work, we consider a dynamic clutter scenario. In this case, when considering multiple measurements, the signaling responses of the low-Q tag have similar properties from one to another. Thus, they present a low-rank structure. Further, the signaling responses of the clutter can be represented as a sparse matrix in the time domain. To this end, we formulate an optimization problem to estimate the low-rank and sparse components from the measurements. This problem is known as robust principal component analysis (RPCA) [15,16]. Note that estimating the sparsest matrix and a low-rank matrix from the observation matrix is an NP-hard problem in which usually convex relaxation of rank and sparsity is utilized. Here, rank and sparsity are replaced with the nuclear norm of a matrix (sum of singular values of a matrix) and $\ell_{1}$-norm of a matrix (absolute sum of elements), which have been well studied in the past [17,18,19,20,21,22,23]. Based on these studies, it is observed that non-convex approaches like reweighted $\ell_{1}$-norm and reweighted nuclear norm minimization have shown better performance compared to the standard convex relaxation.

In many applications, important properties of the data are captured by the large coefficients/singular values of the signal. To capture these properties, it is required to treat the larger coefficients differently from smaller coefficients. Note that standard convex relaxation-based nuclear norm and $\ell_{1}$-norm minimization algorithms shrink all the coefficients/singular values equally. Thus, they are not able to capture important features of the data. However, non-convex approaches like the weighted $\ell_{1}$-norm minimization and weighted nuclear norm minimization algorithms can shrink less the larger coefficients/singular values while weakening the smaller coefficients/singular values [24,25,26,27]. Therefore, in this work, we propose this approach to solve the low-rank plus sparse problem. Also, to estimate the low-rank and sparse components jointly, we propose an iterative algorithm based on the alternating direction method of multipliers (ADMM) [28].

The paper is organized as follows: an overview of the passive localization tags and their operating principles and properties is provided in Section 2. Next, we introduce a vector network analyzer (VNA) based mono-static radar system model for tag identification in Section 3. Section 4 presents the proposed low-rank plus recovery algorithm based on the weighted $\ell_{1}$-norm minimization and weighted nuclear norm minimization for clutter suppression. In Section 5, we provide an evaluation of clutter separation of the low-rank plus sparse recovery approach compared to standard time-gating for the low-Q and high-Q tags. Here, we mainly consider three scenarios, namely free-space, artificial rotating clutter, and real indoor scenario, respectively. Finally, the paper is concluded in Section 6.

## 2. Localization Tag Landmarks

Positioning sensors for indoor/in-room self-localization systems should aim to be passive and cooperative radar landmarks. To do so, the functionalities that they have to provide are two. First, they should comprise a retro-reflective structure, to reflect the incoming interrogation signal to the reader. Second, they must be able to transform their backscattered wave in such a way that each landmark is recognizable and distinguishable by the reader. One possibility to do so is to modify the backscattered frequency response by using a resonating structure, i.e., coding particles. By designing coding particles with different resonance frequencies, several landmarks within a building can be detected and recognized.

Previous works have combined corner reflectors as retro-reflective structures and dielectric resonators [6] or frequency selective surfaces [29] as coding particles, whereas others have used a combination of lenses for long-range reading with cavities implemented in electromagnetic band-gap structures as a combination of retro-reflective and coding parts [9,30]. The aforementioned tags have different operating principles and designs. Thus, to compare them and to achieve a standardized classification, one possibility is to use their quality factor—henceforth referred to as Q-factor.

**Figure 1:**
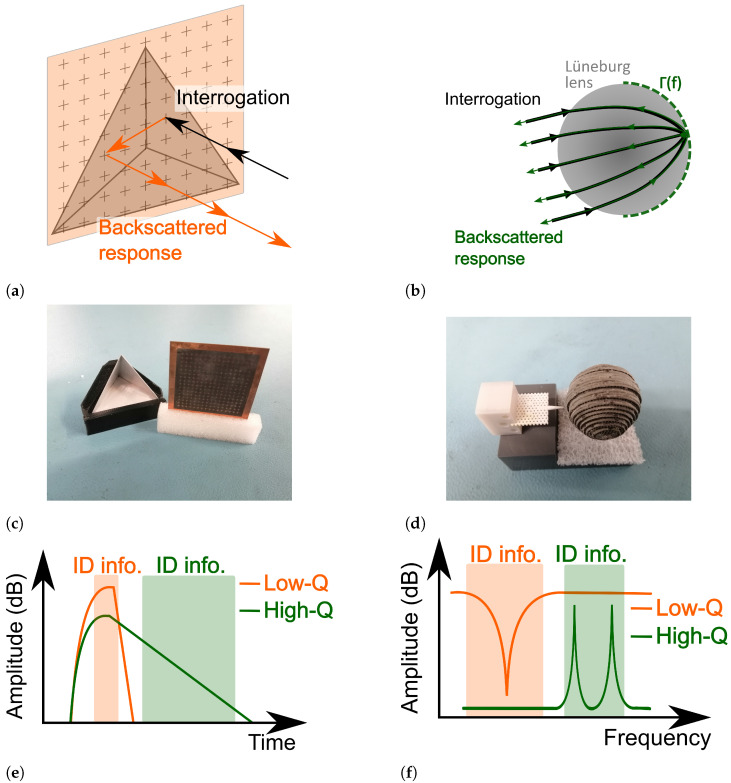
Comparison between low-Q and high-Q tag landmarks. (**a**) Low-Q tag operating principle. The coding part is represented as light orange and the corner reflector as light gray. (**b**) High-Q tag operating principle. The coding particles are represented as green rectangles, whereas the Lüneburg lens is colored gray. (**c**) Low-Q tag used in this paper [29]. (**d**) High-Q tag used in this paper. Combination of [30,31]. (**e**) Backscattered tags’ responses on the time domain. For the low-Q tag, the ID information is encoded in the first received peak, whereas the information of the high-Q is obtained from the low decaying tail. (**f**) Backscattered tags’ responses on frequency domain. The low-Q tag is recognized by having a notch at a concrete frequency, whereas the high-Q tag presents two peaks for ID purposes.

For higher Q-factors, the wave contained within the coding particles re-radiates more slowly back to the reader than for tags with low Q-factors. On the one hand, if the Q-factor is high enough, then the tag’s response can outlast the echoes of the environmental clutter, allowing for its easier detection. On the other hand, low-Q tags are generally structures that are less complex and cheaper to manufacture, but whose responses are more prone to be affected by the surrounding clutter owing to their shorter response time. In this work, clutter-suppression techniques are developed and applied to low-Q and high-Q state-of-the-art landmarks. A brief summary of their operating principles is displayed in Figure 1 for low-Q [29] and high-Q [31] tags, where it should be pointed out that one visible difference is the location of the coding particle.

A sketch of our low-Q tag is displayed in Figure 1a. First, frequency fingerprinting is achieved by using a stop-band frequency selective surface. At resonance, this structure acts as a wall, whereas it is transparent for the frequencies that lie outside its operating bandwidth. Assuming a mono-static radar system, the resonance frequency is reflected away from the receiving antenna (specular reflection), whereas the rest of the spectrum reaches the corner reflector, and thus it is backscattered to the receiver. Therefore, the identification of the landmark is encoded as a notch in the frequency domain, as presented in Figure 1f. Moreover, frequency selective surfaces present low-Q factors, which means that their time response is a short pulse, as displayed in Figure 1e. Finally, a picture of the tag is presented in Figure 1c.

Where low-Q tags first encode the information in the received wave and then reflect it back to the receiver, the high-Q tag presented in this work profits from a photonic crystal-based cavity that performs both. That is, it absorbs an unmodified interrogating wave and re-radiates it slowly towards the reader. Since such structures typically do not have a large backscattered power, its range is increased by using a focusing structure [32], which in our case is a Lüneburg lens. This operating principle is displayed in Figure 1b. Moreover, owing to the high Q-factor of the cavities, sharp resonance peaks are obtained when the tail of the received backscattered response is isolated, as presented in Figure 1e,f. The high-Q tag is presented in Figure 1d, where a supporting structure and a small layer of foam are used to place the tag and lens’ center at the same height.

## 3. System Model

**Figure 2:**
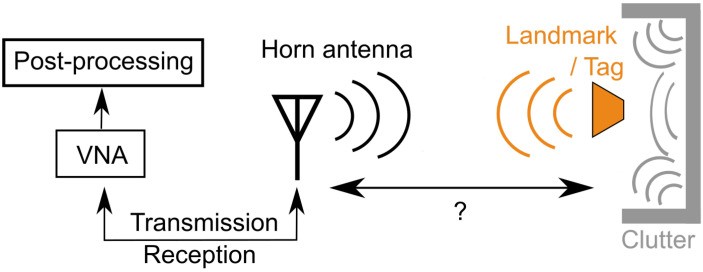
Schematic of the considered mono-static radar scenario.

In this work, we consider a vector network analyzer (VNA) based mono-static mode radar which has a single antenna. Here, we assume that the location of the tag is unknown and it is used as a reference to establish a common coordinate system. To identify the tag, its reflected Electromagnetic wave (EM) signal (backscattered response) is used. In Figure 2, a sketch of the corresponding situation is shown.

The backscattered response at the low-Q tag and high-Q tag have different behaviors, as explained in Section 2. On the one hand, the backscattered response of the low-Q tag is a short pulse in the time domain, and therefore, it has a broad frequency response. On the other hand, the response of a high-Q tag has a long ringing time and hence is sparse in the frequency domain (short pulse). In this work, we profit from the aforementioned properties to separate the tags’ responses from clutter. To this end, the discrete time received signal $y\;\in C^{K}$ is given by

(1) $$y=y_{t}+y_{c}+z.$$

Here, z is the complex additive Gaussian noise. Note that, y consists of two main components, the response of the tag ($y_{t}$) and the clutter ($y_{c}$). Here, our objective is to identify the former from the received signal y. We propose a low-rank plus sparse recovery to separate the desired signal from clutter. However, the received signal y is a vector. Therefore, we consider a workaround to convert received signal y to a matrix to incorporate low-rank property. Here, we generate a Hankel matrix using the received signal y. Now, the received signal matrix after Hankel conversion $Y\;\in C^{M\times N}$ are given by

(2) $$\begin{array}{cc} Y & =H\left(y\right)=H\left(y_{t}\right)+H\left(y_{c}\right)+H\left(z\right)\\ \; & =Y_{t}+Y_{c}+Z. \end{array}$$

The operator $H\left(\cdot \right)$ takes a vector as an input and generates a Hankel matrix. Here, $Y_{t}$, $Y_{c}$ and $Z\;\in C^{M\times N}$ are the received signal matrices of the tag, clutter and noise, respectively. Note that generating a Hankel matrix from a input vector of $x=[x^{1},\cdots ,x^{k}\cdots ,x^{K}]$ is expressed as [33]

(3) $$H\left(x\right)=\left[\begin{array}{cccc} x^{1} & x^{2} & \cdots & x^{K-k+1}\\ x^{2} & x^{3} & \cdots & x^{K-k+2}\\ \vdots & \vdots & \ddots & \vdots \\ x^{k} & x^{k+1} & \cdots & x^{K} \end{array}\right].$$

Here, *k* is the split point and it is also known as the pencil parameter [33]. Further, the inverse Hankel operation denotes as $H^{-1}\left(\cdot \right)$ which takes a matrix as an input and then generates a vector.

Next, we discuss the low-rank plus sparse recovery approach to estimate the received signal matrix of the tag and clutter, respectively.

## 4. Clutter Suppression for High-Q and Low-Q Tags

### 4.1. Low-Rank Plus Sparse Recovery (RPCA) for Clutter Suppression

To retrieve the signal matrixes of tag and clutter, the estimation of $Y_{t}$ and $Y_{c}$ from Y is formulated as a low-rank plus sparsity problem. This problem is known as robust principal component analysis (RPCA) [15,16].

(4) $$\begin{array}{cc} \left\{\hat{Y_{t}},\hat{Y_{c}}\right\} & =\underset{Y_{t},\;Y_{c}}{arg min}\;\lambda_{l}\;\mathrm{rank}\left(Y_{t}\right)+\lambda_{s}{∥Y_{c}∥}_{0}\;,\\ \; & \;\;\;s.t.\;{∥Y-Y_{t}-Y_{c}∥}_{F}^{2}\;\leq \;\epsilon . \end{array}$$

Here, ${\left\|\cdot \right\|}_{0}$ is the $\ell_{0}$-norm of the matrix, and it is given by the number of nonzero elements in the matrix and rank(·) is the rank of the matrix. Note that, $\lambda_{l}$ and $\lambda_{s}$ are given positive regularization parameters. Here, ϵ is a small positive constant (noise bound). The Frobenius norm of a matrix is given by ${\left\|\cdot \right\|}_{F}$. The rank and $\ell_{0}$-norm minimization problems are usually NP-hard. One common approach is considering the convex relaxation, i.e., the nuclear norm of a matrix (sum of singular values) and $\ell_{1}$-norm of a matrix (absolute sum of elements). However, compared to the standard convex relaxation, non-convex approaches like the weighted nuclear norm and weighed $\ell_{1}$-norm have shown improved results [24,27,34,35,36,37]. In this work, we propose weighted nuclear norm and weighed $\ell_{1}$-norm as given below to solve the optimization problem in (4).

(5) $$\begin{array}{cc} \left\{\hat{Y_{t}},\hat{Y_{c}}\right\} & =\underset{Y_{t},\;Y_{c}}{arg min}\;\lambda_{l}\;∥Y_{t}∥{}_{w,🟉}+\lambda_{s}{∥Y_{c}∥}_{w,1},\\ \; & \;\;\;s.t.\;{∥Y-Y_{t}-Y_{c}∥}_{F}^{2}\;\leq \;\epsilon . \end{array}$$

Here, weighted nuclear norm and weighted $\ell_{1}$-norm are denoted by ${\left\|\cdot \right\|}_{w,🟉}$ and ${\left\|\cdot \right\|}_{w,1}$, as given below, respectively.

(6) $$\begin{array}{cc} \|Y_{t}{\|}_{w,🟉} & =\|w_{l}⊙\sigma \left(Y_{t}\right){\|}_{1}, \end{array}$$

(7) $$\begin{array}{cc} \|Y_{c}{\|}_{w,1} & =\|w_{s}⊙Y_{c}{\|}_{1}. \end{array}$$

Here, $w_{l}\in R^{M}$ and $w_{s}\in R^{MN}$ are non-negative weight vectors, respectively. The operator ⊙ denotes element-wise multiplication. Further, $\sigma \left(Y_{t}\right)=\left[\sigma_{1},...\sigma_{i},...,\sigma_{M}\right]\in \;R^{M}$ are the singular values of the matrix $Y_{t}$.

The problem given in (5) is a multi-objective optimization problem, thus the alternating direction method of multipliers (ADMM) is used to solve this problem [28]. Now, we define a Lagrangian function (called augmented Lagrangian function) for the optimization problem is given in Equation (5) as follows.

(8) $$\begin{array}{cc} L(Y_{t},\;Y_{c},\;U) & =\lambda_{l}\;{∥Y_{t}∥}_{w,🟉}+\lambda_{s}{∥Y_{c}∥}_{w,1}+\langle U,Y_{t}+Y_{c}-Y\rangle +\frac{\rho }{2}{∥Y_{t}+Y_{c}-Y∥}_{2}^{2}. \end{array}$$

Here, ρ>0 and $U\;\in C^{M\times N}$ are a penalty factor and an auxiliary variable, respectively. The $\langle \cdot \rangle$ denotes the standard trace inner product. The optimization problem in (8) is solved by alternatively optimizing each component.

Now, let the signal component value at the *t*-th iteration is denoted as ${\left(\right)}^{t}$. To this end, the following optimization problems are solved by alternatively optimizing each component to estimate the $Y_{t}$ and $Y_{c}$. To this end, the value of the $Y_{t}$ at the (t+1)-th iteration is given by

(9) $$\begin{array}{cc} Y_{t}^{t+1} & =\underset{Y_{t}}{arg min}\;\lambda_{l}{∥Y_{t}∥}_{w,🟉}+\frac{\rho }{2}{∥Y_{c}^{t}+Y_{t}-Y+\frac{1}{\rho }U^{t}∥}_{F}^{2}. \end{array}$$

Next, $Y_{c}$ is updated by

(10) $$\begin{array}{cc} Y_{c}^{t+1} & =\underset{Y_{c}}{arg min}\;\lambda_{s}{∥Y_{c}∥}_{w,1}+\frac{\rho }{2}{∥Y_{c}+Y_{t}^{t+1}-Y+\frac{1}{\rho }U^{t}∥}_{F}^{2}. \end{array}$$

Further, U is updated by

(11) $$\begin{array}{cc} U^{t+1} & =U^{t}+\rho \left(Y_{c}^{t+1}+Y_{t}^{t+1}-Y\right). \end{array}$$

The optimization sub-problems given in (9) and (10) are solved in closed-form by using the proximal operators. To be more precise, we utilize the element-wise soft-thresholding and the element-wise singular value soft-thresholding (i.e., element-wise soft-thresholding on the singular value of a matrix) [38,39] as given below.

(12) $$\begin{array}{cc} Y_{t}^{t+1} & ={\mathrm{SVT}}_{\lambda_{LT}}\left(Y-Y_{c}^{t}+\frac{1}{\rho }U^{t}\right). \end{array}$$

(13) $$\begin{array}{cc} Y_{c}^{t+1} & ={\mathrm{ST}}_{\lambda_{ST}}\left(Y-Y_{t}^{t+1}+\frac{1}{\rho }U^{t}\right). \end{array}$$

Here, SVT(·) and ST(·) are the element-wise singular value soft-thresholding and element-wise soft-thresholding operators [38,39], respectively.

Note that $\lambda_{ST}=\left[\lambda_{ST}^{1,1},...,\lambda_{ST}^{m,n},...,\lambda_{ST}^{M,N}\right]$ and $\lambda_{LT}=\left[\lambda_{LT}^{1},...,\lambda_{LT}^{m},...,\lambda_{LT}^{M}\right]$ are the thresholding vectors which contain threshold values for each element of $Y_{c}$ and $\sigma (Y_{t})$, respectively. Here, $\lambda_{ST}$ and $\lambda_{LT}$ change from one iteration to next, however to have a better readability, we drop the iteration index in the notation. Note that the objective of element-wise thresholding is to shrink less large values while shrink more the smaller values. This is because, in many applications, important features and properties are captured in large coefficients/singular values of the signal. Thus, the threshold value should be monotonically decreasing with the coefficient/singular value. Inspired on [24], we consider the log-determinant function to calculate the element-wise threshold values for the t+1-th iteration by utilizing previous estimates of the signals (i.e., $Y_{c}^{t}$ and $Y_{t}^{t}$). To this end, the *m*-th row and *n*-th column element of the $\lambda_{ST}$ and *m*-th element of the $\lambda_{LT}$ are given by

(14) $$\begin{array}{c} \lambda_{ST}^{m,n}=\frac{\lambda_{St}}{\left(\gamma +|y_{m,n}^{c,t}|\right)},\forall m,n. \end{array}$$

(15) $$\begin{array}{c} \lambda_{LT}^{m}=\frac{\lambda_{Lt}}{\left(\gamma +\sigma_{m}^{t}\right)},\forall m. \end{array}$$

Here, γ, $\lambda_{St}$ and $\lambda_{Lt}$ are positive constants. The *m*-th row and *n*-th column element of the $Y_{c}^{t}$ in *t*-th iteration is denoted by $y_{m,n}^{c,t}$. Further, $\sigma_{m}^{t}$ is the *m*-th singular value of the matrix $Y_{t}$ in *i*-th iteration ($Y_{t}^{t}$).

It must be pointed out that this mathematical process can exclusively be applied for the high-Q tag, as its sparse frequency response makes it suitable for such process. However, the low-Q tag features a complementary response, which is sparse on the time domain. Thus, the resulting Hankel matrix is not low-rank, which does not fit with the aforementioned algorithm. To be able to apply RPCA, in the following part a workaround profiting from clutter properties is discussed for this tag.

### 4.2. Low-Rank Plus Sparse Recovery (RPCA) for Low-Q Tag

In contrast with the high-Q tag, the low-Q tag has a broad frequency response. Therefore, the Hankel conversion of the received signal cannot be applied to it, because its backscattered response is not sparse in the frequency domain. Thus, the Hankel matrix generated from the backscattered response of the low-Q tag is not a low-rank matrix. Therefore, we consider a multiple measurement scenario. To this end, we consider there are *Q* number of measurements taken in different incident angles to generate a received signal matrix as given below.

(16) $$Y_{L}=\left[y_{L}^{1},\cdots ,y_{L}^{q}\cdots ,y_{L}^{Q}\right],$$

in which, $y_{L}^{q}\;\in C^{K}$ is the discrete time received signal corresponding to the *q*-th incident angle/measurement. Note that the $y_{L}^{q}\;\in C^{K}$ consists of two components, as given below.

(17) $$y_{L}^{q}=y_{L,t}^{q}+y_{L,c}^{q}+z,$$

where, z is the complex additive Gaussian noise. Note that, $y_{L,t}^{q}$ is the backscattered response of the tag and $y_{L,c}^{q}$ is the clutter. Now, the received signal matrix given in (16) is decomposed as

(18) $$Y^{L}=Y_{t}^{L}+Y_{c}^{L},$$

in which, $Y_{t}^{L}$ and $Y_{c}^{L}$ are the received signal matrices of the low-Q tag and clutter, respectively. The backscattered response of the clutter $y_{L,c}^{q}$ is sparse in the time domain, and hence the matrix $y_{c}$ is a sparse matrix. Also, the backscattered responses of the low-Q tag in different angle positions are similar to each other. Hence, the matrix $Y_{t}^{L}$ is low-rank. To this end, the backscattered response of the low-Q tag $Y_{t}^{L}$ is estimated from $Y_{L}^{L}$ as similar to (4).

(19) $$\begin{array}{cc} \left\{\hat{Y_{t}^{L}},\hat{Y_{c}^{L}}\right\} & =\underset{Y_{t}^{L},\;Y_{c}^{L}}{arg min}\;\lambda_{l}\;\mathrm{rank}\left(Y_{t}^{L}\right)+\lambda_{s}{∥Y_{c}^{L}∥}_{0},\\ \; & \;\;\;s.t.\;{∥Y^{L}-Y_{t}^{L}-Y_{c}^{L}∥}_{F}^{2}\;\leq \;\epsilon . \end{array}$$

The same ADMM based approach described in Section 4.1 is used to solve the optimization problem given in (19).

### 4.3. Parameter Selection for ADMM Based Iterative RPCA Algorithm

Note that in this work, the ADMM based iterative RPCA algorithm given in Equations (9)–(11) is implemented using Matlab [40]. Next, we are going to briefly discuss the effect of the parameters selection in ADMM based RPCA. First, we are going to discuss the selection of the regularization parameters $\lambda_{s}$ and $\lambda_{l}$. Note that the regularization parameters $\lambda_{l}$ and $\lambda_{s}$ balance the two terms $\lambda_{l}{∥Y_{t}∥}_{w,🟉}$ and $\lambda_{s}{∥Y_{c}∥}_{w,1}$ in the optimization problem is given in Equation (5). To explain more clearly, we first modified the optimization problem given in Equation (5) with a single regularization parameter, given as follows

(20) $$\begin{array}{cc} \left\{\hat{Y_{t}},\hat{Y_{c}}\right\} & =\underset{Y_{t},\;Y_{c}}{arg min}\;∥Y_{t}∥{}_{w,🟉}+\frac{\lambda_{s}}{\lambda_{l}}{∥Y_{c}∥}_{w,1},\\ \; & \;\;\;s.t.\;{∥Y-Y_{t}-Y_{c}∥}_{F}^{2}\;\leq \;\epsilon . \end{array}$$

Now, the regularization parameter corresponding to the low-rank component $Y_{t}$ is 1 and let it is denoted by $\lambda_{L}$, and now $\lambda_{L}=1$. Let consider $\lambda_{S}=\lambda_{s}/\lambda_{l}$. When $\lambda_{S}\to 0$, the sparsity of the recovered sparse matrix $\hat{Y_{c}}$ becomes irrelevant and $\hat{Y_{c}}$ approaches the receive signal Y. That is, the estimated clutter $\hat{Y_{c}}$ is very similar to the received signal. Then, the recovered low-rank matrix $\hat{Y_{t}}$ (i.e., tag’s response) tends to be a zero matrix, i.e., we are unable to recover the tag’s response. On the other hand, when $\lambda_{S}\to \infty$, the recovered sparse matrix $\hat{Y_{c}}$ (estimated clutter) tends to be a zero matrix. Thus, $\lambda_{S}$ should not be very small or very large. It is important to select appropriate values for the $\lambda_{L}$ and $\lambda_{S}$ which replicates the correct balance between the low-rank and the sparse components. Further, as suggested by [15], when RPCA problem is solved by the $\ell_{1}$-norm minimization and nuclear norm minimization, as a rule, $\lambda_{S}$ is chosen as $\lambda_{S}=1/\sqrt{\max(M,N)}$. However, in this work, we propose weighted nuclear norm and weighed $\ell_{1}$-norm minimization to solve the RPCA problem. Therefore, initial values of $\lambda_{L}$ and $\lambda_{S}$ are set to choose as 1 and $1/\sqrt{\max(M,N)}$, respectively. Then, their values are tuned based on the data. To the best of our knowledge there is no specific rule to select $\lambda_{S}$ and $\lambda_{L}$ when RPCA problem is solved by using the weighted nuclear norm and weighed $\ell_{1}$-norm minimization. Note that, the singular value soft-thresholding ($\lambda_{Lt}$) and soft-thresholding parameters ($\lambda_{St}$) given in (14) and (15) are set as $\lambda_{L}/\rho$ and $\lambda_{S}/\rho$, respectively. Here, ρ is the ADMM penalty factor given in (8).

Next, we discuss how to select the noise bound (error tolerance) given in Equation (5). If we set the error tolerance ϵ to a too low value, we are unable to recover the low-rank and sparse matrices within the error bound. If we set it to a too high value, the combination of the recovered low-rank and sparse matrices ($\hat{Y_{t}}+\hat{Y_{c}}$) drifts away from the Y. In this work, we observed that $\epsilon =10^{-5}$ is a suitable value for our data. The ADMM penalty factor ρ given in (8) plays an important role. Theoretically, if ρ is relatively large the algorithm converges faster. However, finding the best value for ρ is an open problem. It is shown in [16] when ρ is very small or very large, the ADMM-based RPCA algorithm requires more iterations to achieve satisfactory recovery errors for the recovered low-rank and sparse matrices. Therefore, as a rule, ρ should not be very small or very large. Note that selecting the best ρ is problem-specific, yet as a rule of thumb, ρ can be selected as $0.25MN/{∥Y∥}_{1}$ [16]. However, for our work, it is observed that $\rho =0.25MN/{∥Y∥}_{1}$ is too large and thus we set a smaller value for ρ as $\rho =10/\sqrt{\max(M,N)}$.

The positive constant γ is given in Equations (14) and (15) are used to enforce numerical stability of the algorithm. In more details, the purpose of the positive constant γ to enforce stability when $\sigma_{m}^{t}$ or $y_{m,n}^{c,t}$ is zero [24]. Note that γ should be set slightly less than the expected non-zero values of $Y_{c}$ and non-zero singular value of the matrix $Y_{t}$. In this work, we set γ=1 since it is slightly less than the expected non-zero values of the $Y_{c}$ and non-zero singular values of the matrix $Y_{t}$. Further, the maximum number of iterations of the ADMM based iterative RPCA algorithm is set to 500. The algorithm is terminated if it reaches the maximum number of iteration or the reconstruction error ${∥Y-Y_{t}-Y_{c}∥}_{F}^{2}$ given in (5) drops below $10^{-5}$, i.e., $\epsilon =10^{-5}$.

## 5. Measurement Results and Discussion

In this section, we analyze the performance of RPCA when separating the tags’ backscattered responses from surrounding clutter. The obtained signals are compared with the result of using time-gating, a classical clutter-separating technique that consists in the isolation of the wanted signal between two concrete time steps, leaving the surrounding clutter outside the time-gated interval. This technique –albeit very powerful– assumes a-priory knowledge of the distance between the tag and interrogating antenna to isolate the former, which is a drawback in an indoor self-localization scenario, where the landmarks’ locations might be unknown.

All measurements are performed using a Keysight Technologies N5222A vector network analyzer (VNA) along with an Anritsu 3740A extension connected to a standard 25 dBi W-Band horn antenna, that acts as a mono-static radar system. The separation between the low-Q and high-Q tags and the antenna is set to 80 cm, which guarantees that both tags are within the far-field of the latter. The wave emitted by the horn antenna is vertically polarized.

The order of this section is as follows. Firstly, free-space results are displayed to compare the performance of time-gating and RPCA in equal conditions, as well as validating the latter for tag identification. Secondly, the tag is located within an artificially designed cluttered scenario and the validity of the clutter separation algorithms is addressed by comparing their extracted responses. Thirdly, a preliminary measurement of the landmarks is presented in a real indoor scenario. In this case, the corner of a laboratory room.

### 5.1. Low-Q Landmark

As presented in Figure 1, the low-Q tag presents a notch at its operating frequency. Concretely, it lies at 77 GHz [29], with a relative bandwidth of 10%.

#### 5.1.1. Characterization

**Figure 3:**
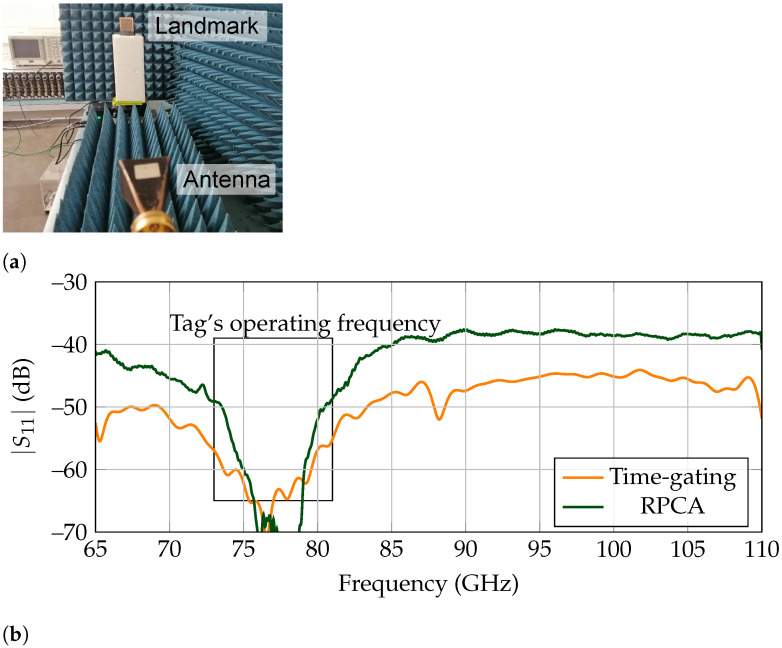
Free-space measurement for low-Q landmark. (**a**) Measurement set-up. (**b**) Backscattered frequency response.

To achieve free-space measurements, the tag is surrounded by absorbers to prevent reflections from the surrounding media and located in a supporting foam, as presented in Figure 3a. It is noteworthy that the tag is rotated 20, regarding its axis. Otherwise, in the case of frontal incidence between the tag and reader, the combination of backscattered response of the corner reflector and specular reflection of the frequency selective surface results in an added complexity when detecting the tag’s response [41].

The frequency response results for the free-space measurement are presented in Figure 3b. To achieve a result as accurately as possible, first an empty room measurement is performed and subtracted to the measured tag results. Time-gating is performed between 5.85 ns to 6.85 ns to leave out a standing wave between antenna and landmark. It is noticeable that RPCA can also extract the correct response, which validates its usage for the detection of such structures.

#### 5.1.2. Cluttered Measurements

**Figure 4:**
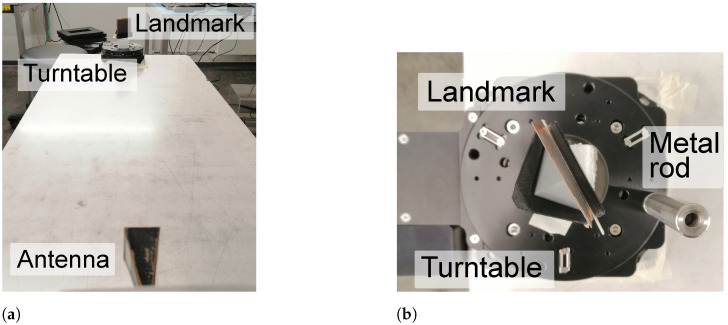
Cluttered set-up for low-Q landmark. (**a**) Clutter set-up for low-Q tag. (**b**) Closer view on the moving clutter set-up.

The performance of time-gating and RPCA regarding clutter is evaluated in the measurement set-up presented in Figure 4a. As it can be identified from the image, there are mainly four clutter sources present:

- The early reflections of the antenna, resulting of a mismatch between the horn antenna and the WR10 extension.
- Reflections on the “ground” i.e., the table that supports the set-up.
- Reflection on the measurement turn-table, which is completely made up of metal.
- Late environmental clutter owing to the surrounding laboratory room, such as reflection on columns, equipment, or walls.

Moreover, a small metallic cylinder is located 3 cm away from the tag and is rotated around it, to simulate a small moving object. The dimensions of the cylinder are a diameter of 1.2 cm and a height of 8.5 cm. A closer image of this setup is displayed in Figure 4b.

**Figure 5:**
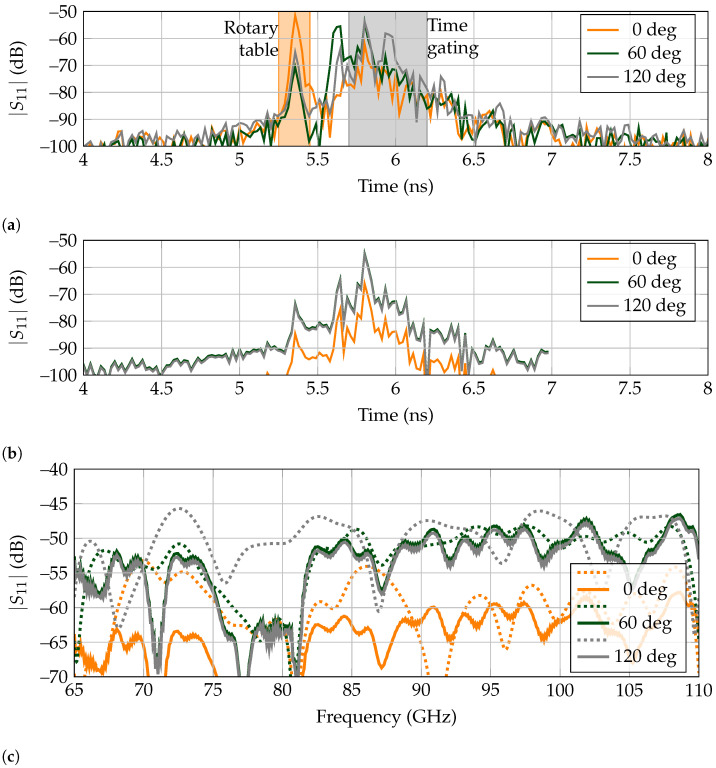
Selected backscattered responses for the low-Q tag when encircled by a small metallic rod. (**a**) Measured time responses. Time gating is marked in gray and spans from 5.7 ns to 6.2 ns. The early reflection of the rotary table is marked in orange. (**b**) Extracted time-domain backscatter response of the tag by the RPCA. (**c**) Frequency domain of extracted signals. Time gating results are represented by dashed lines, whereas RPCA by the solid lines.

In Figure 5a, the received signal are displayed when the metal rod presented on Figure 4b is rotated to the positions of 0, 60 and 120 regarding the direct line-of-sight between antenna and landmark. It is noticeable that for all of them, there is a first received peak at 5.35 ns, corresponding to the reflected power by the turntable. The peak is noticeably higher for the angle of 0as the metal rod is located between the tag and antenna, at the same position as the turntable. Thus, it contributes to the higher amplitude of the received backscattered power. Afterward, for 60 there is a high amplitude peak at 5.6 ns, corresponding to the metallic rod. It is appreciated that it lies outside the time-gating window, so the landmark’s response is received and isolated correctly. Then, the echo resulting from the landmark is received at 5.80 ns. Finally, for 120, there is a high amplitude peak at 5.95 ns, corresponding to the metallic rod being behind the tag. As this received signal is contained within the selected time-gating span, no separation is carried out between the clutter and landmark’s responses, receiving a signal where the detection of the tag is difficult.

The results in the frequency domain for the time-gated responses, presented as dashed lines in Figure 5c, further confirm the previous asseveration. First, for 0 there is a notch at the operation frequency of 77 GHz, but the rest of the bandwidth also presents a rippling response, with very low amplitude at most frequencies, owing to the blockage by the metallic rod. Second, the received signal at 60 is very similar to the free-space measurement presented in Figure 3b, which means that the landmark’s response is successfully separated from clutter. Third, the received response at 120 does not present a deep notch at 77 GHz, since the metallic rod echo is present in the time-gated results. Although it is possible to recognize a small notch at the corresponding frequency, it is owing to the landmark being present between the antenna and rod, so it is first reached by the interrogating wave. Presumably, in the case that the clutter was a larger object instead of a thin metallic rod, its echo could potentially mask the tag’s backscattered response.

In Figure 5b, the extracted tag responses are presented for the angular rotations of 0, 60 and 120 by RPCA. It is noticeable that the turntable echo at 5.6 ns is very attenuated for the three angles, as it has been extracted as clutter. The same situation happens for the metallic rod present for 60and 120, which is completely suppressed. Thus, with the exception of when the rod is in the direct line-of-sight between tag and reader, the latter response is always properly received. The results in the frequency domain for RPCA, presented as solid lines on Figure 5c, showcase that the tag is detected even at 120, which was not possible with time-gating.

**Figure 6:**
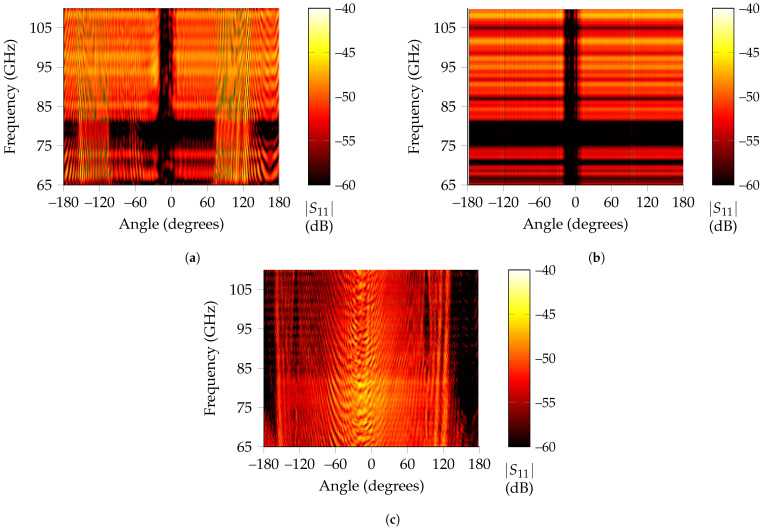
Received power in terms of angle and frequency when a small metallic rod rotates around the low-Q tag. (**a**) Time-gating results for all angles. The two received high-amplitude regions on the sides, owing to the rod encroaching on the time-gated span, and the deep notch region on the center due to blockage are easily noticeable. (**b**) RPCA extracted tag responses for all rotations. Only the low received amplitude region remains. (**c**) Clutter extracted by RPCA. It is noticeable the high amplitude response around blockage area (Approx. from –30° to 0°).

To give an extended overview of the performance of RPCA, the received signal is analyzed when the metallic cylinder completes a whole loop around the tag. The resulting heatmaps that present the received signal regarding each angle can be found in Figure 6a for time-gating and on Figure 6b for RPCA.

On the one hand, from the time-gated results, three angular regions stand out above the rest, namely from –155 to –105, –19 to +4 and +70 to +143. The first and third of them present a very high backscattered amplitude and correspond to the situations where the metallic rod is close enough to the tag that it encroaches in the time-gating window, so no separation between both responses is possible. The second one is a notch, corresponding to the angles in which the rod is between the line-of-sight path of the antenna and landmark. On the other hand, the RPCA algorithm can achieve the correct landmark response for all angles but for line-of-sight situations, where blockage of the metallic rod difficulties successful tag recognition.

In Figure 6c, the extracted response for clutter is displayed, where it is noticeable its higher amplitude for rotations that involve line-of-sight blockage because the metallic rod reflects most of the EM waves at those angles. Nevertheless, as presented in Figure 5c, it is still possible to recognize a notch at the landmark’s operating frequency for these angles, while the rest of the spectrum is attenuated 10 dB on average owing to the relatively large size of the tag compared to the metallic rod. The diameter of the metallic rod is 1.2 cm, whereas the edge of the corner reflector used is 3 cm. This means that, even for line-of-sight blockage, the tag is still backscattering a frequency-coded wave, albeit with a very attenuated amplitude.

#### 5.1.3. Indoor Scenario

**Figure 7:**
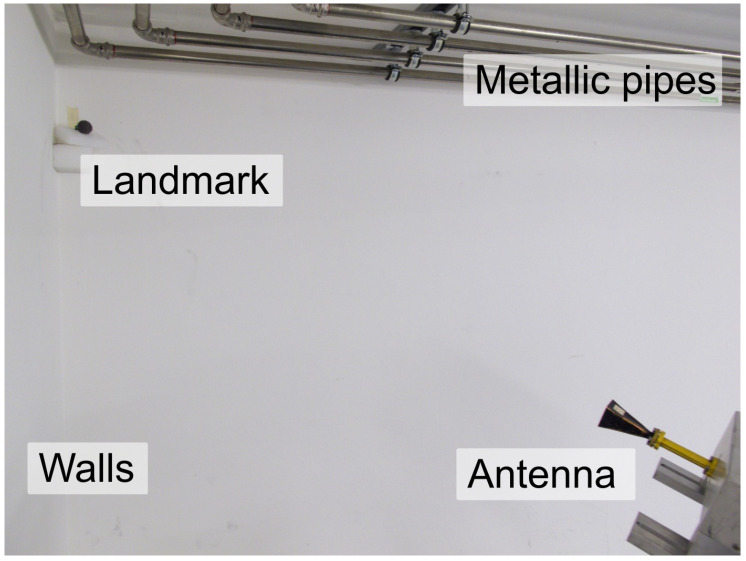
Real indoor set-up for low-Q and high-Q landmarks. Only the latter appears on this image.

In light of the above, the RPCA algorithm can perform better than conventional time-gating in a controlled environment for low-Q tags. As a first evaluation in a real indoor environment, we placed the landmark in the corner of the laboratory, where the clutter can be considered to be caused by the walls, conforming a natural trihedral reflector, as well as the pipes present in Figure 7. The distance between the antenna and landmark is set to 1 m in this case. Moreover, the backscattered wave is time-gated between 4 ns to 12 ns to leave out the early reflections of the antenna for clarity, as it has been shown in the previous sections that it does not affect the ability to successfully retrieve the landmark’s response.

**Figure 8:**
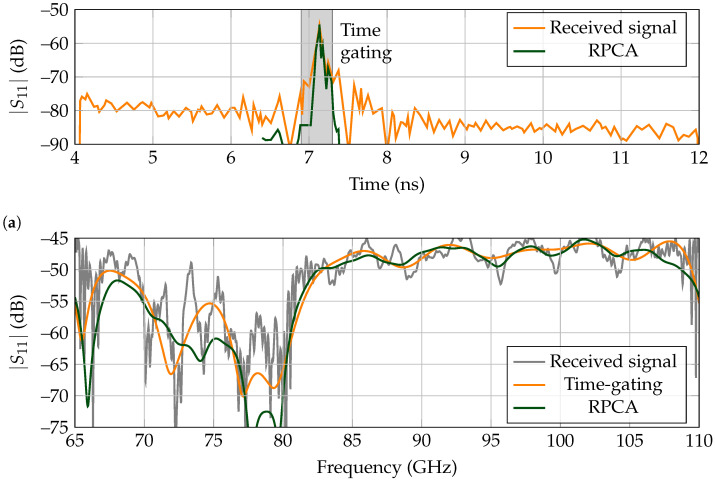
Backscattered results for a low-Q landmark for measurements performed in a corner of the laboratory. (**a**) Time response. Time gating is performed between 6.9 ns to 7.3 ns. (**b**) Frequency response.

The received backscattered wave in the time domain is presented in Figure 8a, whereas the frequency response is displayed in Figure 8b. For this case, both the time-gating and RPCA can retrieve the landmark’s response, although the small window span used for the time-gating approach should be pointed out, of 400 ps.

### 5.2. High-Q Landmark

As displayed in Figure 1, the high-Q tag presents a peak at its operating frequency. Concretely, the tag used in this work presents two resonance frequencies at 71.61 GHz and 72.32 GHz [31].

#### 5.2.1. Characterization

**Figure 9:**
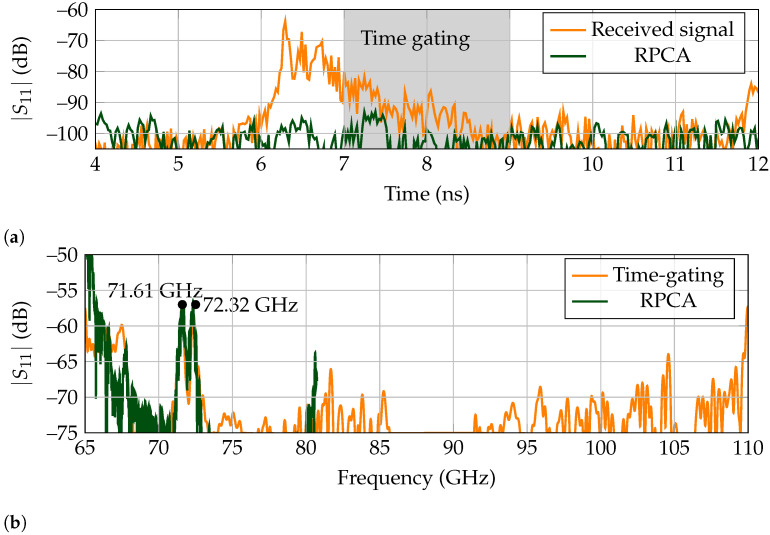
Free-space measurement for high-Q landmark. (**a**) Received time response. (**b**) Backscattered frequency response. The raw received signal is not shown.

The set-up used to measure the free-space response of the high-Q tag is the same as presented in Figure 3a. The frequency response results for the free-space measurement are presented in Figure 9b. To achieve a result as accurately as possible, first an empty room measurement is performed and subtracted to the measured tag results. As the identification information of the high-Q tag is encoded on the tail of its backscattered response, time-gating is performed between 7 ns to 9 ns. It should be pointed out that RPCA and time-gating present similar results, since the measurements are performed in a free-space scenario. That is, there is no clutter present that can affect the extracted response.

#### 5.2.2. Cluttered Measurements

**Figure 10:**
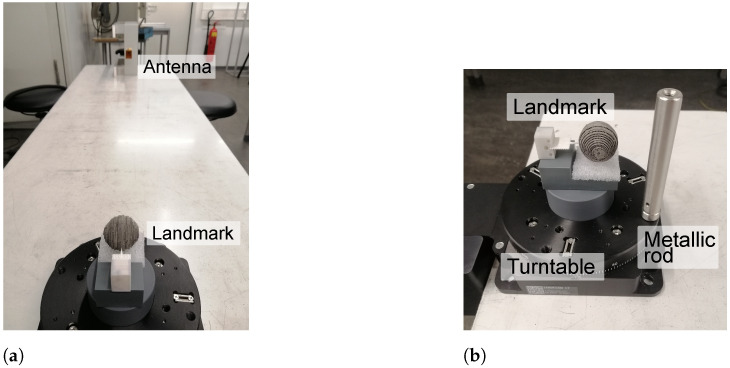
Cluttered set-up for high-Q landmark. (**a**) Clutter set-up for high-Q tag. (**b**) Closer view on the moving clutter set-up.

The measurement set-up to evaluate the performance of time-gating and RPCA is presented in Figure 10a. In comparison with the low-Q tag, there is one more clutter source present. That is, the reflection of the landmark itself. As the frequency fingerprinting is encoded on the tail of its backscattered response, the first specular reflection of the tag is also undesired for identification purposes. The set-up with the small metallic rod is very similar to the one presented for the low-Q landmark, with the exception that the rod is located so that it starts at approximately 0, blocking the line-of-sight between landmark and antenna.

**Figure 11:**
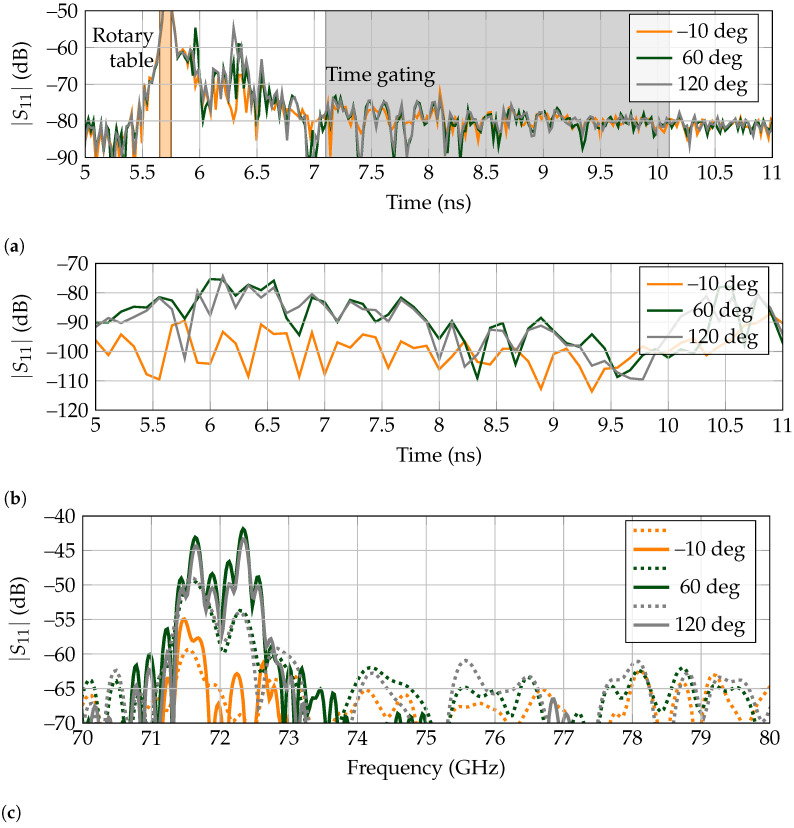
Selected backscattered responses for the high-Q tag in the cluttered scenario. (**a**) Measured time responses. Time gating is marked in gray and spans from 7.1 ns to 10.1 ns. The early reflection of the rotary table is marked in orange. (**b**) Extracted time-domain backscatter response of the tag by the RPCA. (**c**) Received frequency response, where dashed lines indicate time-gating and solid lines RPCA. The plot is represented between 70 GHz to 80 GHz to facilitate comparison at resonance.

In Figure 11a, the received signals are displayed when the metal rod presented in Figure 10b is rotated to the positions of –10, 60, and 120 regarding the direct line-of-sight between antenna and landmark. As it was shown in Figure 9b that there are no other high amplitude peaks in the spectrum but the tag’s resonance frequencies, the results are represented from 70 GHz to 80 GHz. The time-gating is performed between 7.1 ns and 10.1 ns, completely separating the received signal from the turntable and metallic rod echos, as the tail of the tag’s backscattered response outlasts the surrounding clutter. For 60 and 120 the frequency response of the tag is identified unequivocally, with the RPCA-extracted results presenting an increased in backscattered power regarding time-gating of approx. 5 dB for the resonance at 71.61 GHz and of approx. 12 dB for the second resonance at 72.32 GHz as shown in Figure 11c. Since the detection of both resonances is needed for identification, this 12 dB increase in amplitude can be considered as an identification gain that, following the range equation for monostatic radars, increases the range by two, from 80 cm to 160 cm. However, the performance of RPCA depends on the dynamic range of the reader. Thus, this statement must be verified experimentally. Finally, for –10 both time-gating and RPCA fail to correctly extract the landmark’s response, owing to the blockage caused by the metallic rod.

**Figure 12:**
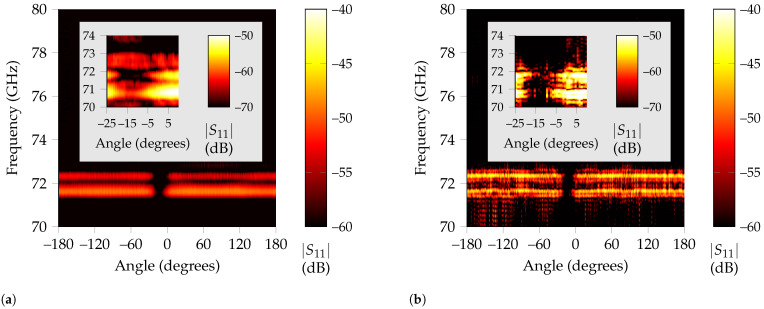
Received power in terms of angle and frequency when a small metallic rod rotates around the high-Q tag. (**a**) Time-gating results for all angles. The deep notch region on the center due to blockage is easily noticeable. (**b**) RPCA extracted tag responses for all rotations. The tag’s resonances present a larger power than the time-gating results.

To analyze blockage in detail, in Figure 12a,b the corresponding received power for each angle is represented. As expected, the only angular region where the identification of the landmark is more challenging is when the metallic rod blocks the line of sight. Although this region seems to span from –20 to 5, when a zoom-in to that area is performed and the scale adjusted accordingly, it is noticeable that there are several angles included in this region for which the tag can be successfully detected, albeit with lower amplitude. The decrease in received power regarding the rest of the angles is because some of it is reflected on the metal rod, which in turn decreases the intensity of the EM wave that reaches the tag. Finally, for the region between –5 to –15 the blockage is complete and no identification of the resonances is possible. Note that, in the zoomed region of Figure 12, time-gating also presents an undesired peak around 73 GHz. However, for RPCA this is not the case.

#### 5.2.3. Indoor Scenario

In light of the above, the RPCA algorithm performs slightly better than conventional time-gating in a controlled environment for high-Q tags. As with the low-Q landmark, we placed the tag in a corner of the laboratory, as presented in Figure 7. The distance between the antenna and landmark is set to 1 m. Finally, the backscattered response is time-gated between 4 ns to 12 ns to leave out the early reflections of the antenna for clarity.

**Figure 13:**
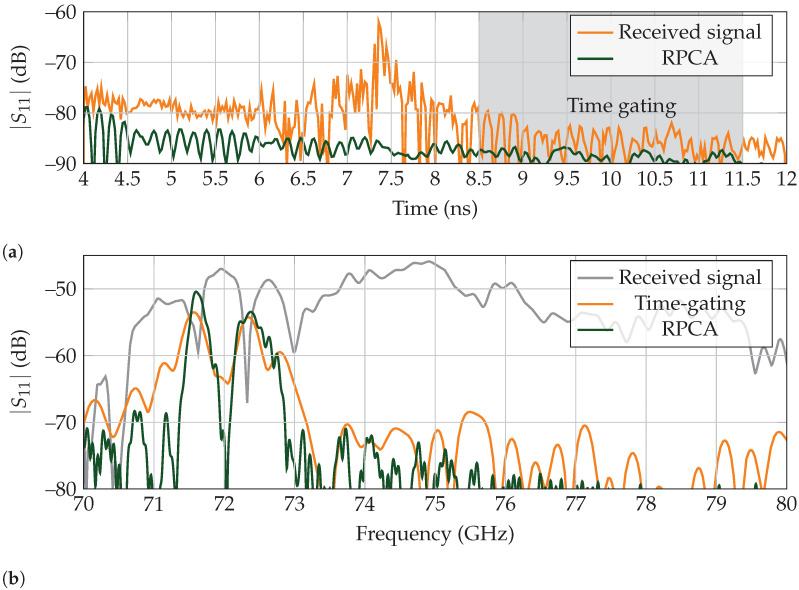
Received backscattered results for a high-Q landmark for measurements performed in a corner of the laboratory. (**a**) Time response. Time gating is performed between 8.5 ns to 11.5 ns. (**b**) Frequency response.

The received backscattered wave in the time domain is presented in Figure 13a, whereas the frequency response is displayed in Figure 13b. Although both clutter-separating techniques can retrieve the tag’s response, RPCA presents an improvement on the notch depth between the identification resonances at 71.61 GHz and 72.32 GHz of approximately 10 dB, which eases their correct separation. Moreover, the rest of the spectrum is also suppressed by 10 dB, compared to the time-gated results. Thus, identification of the tag when applying RPCA is expected to be more reliable than time-gating.

## 6. Conclusions

In this work, we have evaluated the performance of state-of-the-art clutter suppression methods, namely time gating and low-rank-plus sparse recovery/robust principal component analysis (RPCA). Note that in this work we use the reweighted nuclear norm and reweighted $\ell_{1}$-norm in RPCA instead of the standard nuclear-norm and $\ell_{1}$-norm. Both algorithms are applied to the identification of two landmarks for indoor self-localization applications with complementary operating principles. Based on our results, it is shown that RPCA can outperform time-gating when separating the response of a low-Q tag from clutter, being able to retrieve the tag’s identification even in the case of very close clutter, mostly owing to the difficulties of selecting a correct time window for all measurements. RPCA has shown significant improvements for the high-Q tags, achieving an identification gain of 12 dB for a controlled cluttered scenario and a suppression of 10 dB for the outside-of-resonance spectrum, in a specific real-world environment.

There are several possibilities for future work, orientated to confirm these preliminary results about the potential of RPCA in cluttered environments in millimeter-wave. First, measurements could be carried out with a frequency modulated continuous wave radar, which mimics the functionality of a VNA in the sense that it can receive a complex signal that can be used to apply clutter-separating algorithms, but with a lower cost and footprint. Second, machine learning could be considered to learn the parameters of the RPCA algorithm (i.e., thresholding parameters), with the objective to increase its efficiency, convergence rate, and robustness. This is a promising area in signal processing, known as algorithm unrolling. Third, the real-time application should be considered by accelerating the RPCA algorithm using FPGAs, as it has been demonstrated for synthetic aperture radars on [42]. Finally, more complex and dynamic scenarios with objects of various sizes and moving around should be considered, as well as the performance of RPCA in multi-tag scenarios.

## Data Availability

The data can be provided by the authors J.S.-P. or U.S.K.P.M.T. upon reasonable request.
